# Valerianate of Ammonia in the Treatment of Neuralgia

**Published:** 1857-10

**Authors:** W. Hay

**Affiliations:** Chicago


### ARTICLE II.— *Valerianate of Ammonia in the Treatment of Neuralgia.* By W. Hay, M. D., Chicago.

EXPERIMENT NO 1.

*June* 12*th*, 1857.—Patient, a lady laboring under incipient tuberculosis and general anaemia, was seized with severe neuralgic pains in the right temple, extending over the entire half of the head and face. Local applications of Chloroform were made, which mitigated the pains considerably, but did not relieve them entirely until after the lapse of five or six hours.

*June* 13, 14, 15, 16, 17, 18.—The pain still persistent.

*June* 19*th*.—Directed the following:

℞.

Tinct. Valerian Ammoniate, ʒ vj

Tinct. Opii, ʒ ij

M

One teaspoonful to be taken every three hours, local applications to be continued. This treatment was persisted in for several days with no perceptible effect, beyond the production of great irritability of stomach with distressing nausea and vomiting; it was therefore discontinued, and the following was directed:

℞. Tinct. Aconite.

To be applied externally, and given internally in doses of five drops every two hours. This was continued twenty-four hours without producing any benefit. She had now become so intense, the paroxysms being greatly lengthened, and the intermissions proportionally shortened, as to necessitate a return to the Chloroform applications, together with its exhibition by inhalation. Thinking that some local cause might be at the bottom of the difficulty, had the patient removed to a much higher and more airy residence, on the 3d of July.

*July* 4*th*.— Pain continuing with very slight intermissions. Directed ʒ j Ferri. Sub. Carb. Praecip. to be taken every four hours, alternated with five grs. Bismuth Sub Nitrate. The nausea was by this means relieved, the pain ameliorated for two or three hours only, returning after that interval with increased violence.

*July 6th.*—Directed ten drops Sol. Potass. Arsenic, together with a scruple of Tinct. Cannabis. Indic. to be given every six hours, which was attended with no benefit whatever.

*July 7th.*—Patient suffering acutely, to take the following:

℞.

Ammoniae Valerianta (deliquescent) ʒ j

Aqua. ℥ j

Dose, one teaspoonfull. One dose was taken at 7 P. M., and was followed by a marked decrease of pain, increased force and diminished frequency of pulse. On the administration of the second dose at ten o'clock, P. M., the patient sunk into a quiet sleep, in which she continued until morning.

*July 8th.*—To continue the treatment, as on last night, pain much diminished, but not entirely gone.

*July 9th.*— Continue treatment.

*July 10th.*— Continue treatment.

*July 14th.*—By this time the salt seemed to have lost its controlling power over the disease. The dose was doubled, but produced only distressing dyspnœa, and head-ache. The pain was still persistent. The treatment was, therefore, abandoned, and the patient directed to take ʒ ss. Ferri. Sub. Carb. Praecip. The first dose was followed by immediate and permanent relief from pain. This treatment was pursued for some days, as a precautionary measure, but the pain did not return.

My attention was first directed to the Valerianate of Ammonia in the treatment of Neuralgia, from reading the report of a case successfully treated with this salt, by Dr. Declat of Paris, contained in the last vol. of Ranking Half-Yearly Abstract. It would be proper to remark here, that an idiosyncracy of this patient contra indicating the use of any of the vegetable alkaloids, rendered it necessary to seek relief, in some agent out of the usual category of remedies. I would also state that the specimen of Valerianate of Ammonia used in this case, was of doubtful strength, having absorbed so much water as to be perfectly liquid.

EXPERIMENT NO. 2.

*August 19th*, 1857.—The same patient, as above, was attacked about midnight, with Neuralgia in the right side of the head and face, as before, and also in the intercostal nerves of the same side. The pain was so severe as to obstruct respiration to a very serious extent, the functions of the right lung being already impaired by pre-existing disease. Such remedies as were at hand were used during the night, entirely in vain, temporary suspension of the pain being obtained by the inhalation of Chloroform.

*August 20th.*—Pain returned at noon after an interval of about twelve hours, and continued with great severity during the remainder of the day. Being desirous to test fully and fairly the powers of the Valerianate of Ammonia, I procured with some difficulty a small quantity in the crystaline state, and administered one grain of the salt in a teaspoonful of water, at 10 o'clock P. M., this was followed by almost immediate relief from pain, and, as in the former case, by increased force and diminished frequency of the pulse; in fifteen minutes the patient was asleep.

Relief in this case has been permanent.

